# Identification of HOXB9 to predict prognosis of endometrial cancer based on comprehensive bioinformatics analysis

**DOI:** 10.1186/s40001-022-00979-3

**Published:** 2023-02-17

**Authors:** Yanhua Xu, Mu Zhang, Qin Shi, Xi Cheng, Rong Du, Chenglu Li, Yuquan Zhang

**Affiliations:** 1 Department of Obstetrics and Gynecology, Affiliated Hospital of Nantong University, Medical School of Nantong University, No.20 Xi-Si Road, Nantong, 226001 Jiangsu China; 2 Department of Ophthalmology, Affiliated Hospital of Nantong University, Nantong, 226001 Jiangsu China; 3 Center For Reproductive Medicine, Affiliated Hospital of Nantong University, Nantong, 226001 Jiangsu China

**Keywords:** Endometrial cancer, HOXB9, TCGA, Bio-informatics analysis, Prognosis, Nomogram

## Abstract

**Background:**

The HOXB9 gene, which plays a key role in embryonic development, is also involved in the regulation of various human cancers. However, the potential relationship between HOXB9 and endometrial cancer (EC) has not yet been comprehensively analyzed and fully understood.

**Methods:**

We used multiple bioinformatics tools to explore the role of HOXB9 in EC.

**Results:**

The expression of HOXB9 was significantly upregulated in pan-cancer, including EC (*P* < *0.05*). Quantitative real time polymerase chain reaction (qRT-PCR) experiment confirmed the high expression of HOXB9 in EC from clinical samples (*P* < *0.001*). Double validated by Enrichr and Metascape, HOXB9 showed a strong correlation with HOX family, suggesting that HOX family may also involve in the development of EC (*P* < *0.05*). Enrichment analysis revealed HOXB9 is mainly associated with cellular process, developmental process, P53 signaling pathway, etc. At the single-cell level, the clusters of cells ranked were glandular and luminal cells c-24, glandular and luminal cells c-9, endothelial cells c-15, compared with the other cells. At the genetic level, promoter methylation levels of HOXB9 were significantly higher in tumors than in normal tissues. Furthermore, variations of HOXB9 were closely associated with overall survival (OS) and recurrence free survival (RFS) in EC patients (*P* < *0.05*). The agreement between univariate and multivariate Cox regression indicated that the results were more reliable. Stages III and IV, G2 and G3, tumor invasion ≥ 50%, mixed or serous histological type, age > 60 years, and high expression of HOXB9 were risk factors strongly associated with OS in EC patients (*P* < *0.05*). Therefore, six factors were incorporated to construct a nomogram for survival prediction. Finally, we used the Kaplan-Meier (KM) curve, receiver operating characteristic (ROC) curve, and time-dependent ROC to assess predictive power of HOXB9. KM curve showed EC patients overexpressing HOXB9 had a worse OS. AUC of diagnostic ROC was 0.880. AUCs of time-dependent ROC were 0.602, 0.591, and 0.706 for 1-year, 5-year, and 10-year survival probabilities (*P* < *0.001*).

**Conclusions:**

Our study provids new insights into the diagnosis and prognosis of HOXB9 in EC and constructs a model that can accurately predict the prognosis of EC.

**Supplementary Information:**

The online version contains supplementary material available at 10.1186/s40001-022-00979-3.

## Background

Endometrial cancer (EC) is one of the most common gynecologic tumors in obstetrics and gynecology [1]. In the United States, EC currently affects approximately 62,000 women and causes more than 12,000 deaths annually [2]. In China, the number of patients with EC was approximately 69,000, the number of deaths was 16,000, and the incidence rate was 10.28/100,000, accounting for 3.88% of malignant tumors in women [3]. The primary treatment for EC is surgery and depends largely on the stage of the disease [4]. Most patients with early stage EC have better outcome after surgical resection [5]. However, it has been shown that postoperative recurrence is the main cause of increased mortality. Although traditional clinical features, including tumor grade, FIGO stage, histologic type, and lymphatic metastasis are currently considered as risk factors, they cannot accurately predict the prognosis of EC. Therefore, identifying the best predictive prognostic factors for EC is key to clinical research.

The HOX gene encodes a set of transcription factors that share a highly conserved homologous box domain. In vertebrates, all 39 HOX genes have been identified and divided into four clusters (HOX-A, B, C, and D) [6–8], which are located on four different chromosomes (7p15, 17p21, 12q13, and 2q3) [9, 10]. Together with HOX genes, HOXB9 controls the skeletal elements of the thoracic cage and development of the mammary glands [11, 12]. Apart from playing a key role in embryonic development, HOXB9 is involved in the regulation of various human cancers [13, 14]. Wan et al. discovered HOXB9 regulates the progression of lung adenocarcinoma by directly targeting JMJD6 [14]. Additionally, up-regulation of HOXB9 results in poor overall survival in many cancer patients and promotes epithelial–mesenchymal transformation [15–17]. However, the expression and specific function of HOXB9 in EC remain unclear.

In this study, we systematically analyzed the expression and regulatory network of HOXB9 and HOX family in EC using pan-cancer analysis, clinicopathological parameters, co-expression, Gene Ontology (GO) and Kyoto Encyclopedia of Genes and Genomes (KEGG) enrichment analysis, protein-protein interaction (PPI) network, single-cell RNAseq, DNA methylation, HOXB9 gene alterations, etc. Then, we constructed a prognosis-related nomogram based on the results of univariate and multifactorial Cox regression analysis, and validated it using KM curves and ROC. Interestingly, high expression of HOXB9 and its variants were closely associated with OS, RFS in EC patients. Our results showed HOXB9 plays a crucial role in EC, and nomogram can accurately predict the prognosis of EC, which may provide new insights into its clinical management.

## Materials and methods

### Pan-cancer analysis

TIMER2.0 (http://timer.cistrome.org/) [18] is a comprehensive resource for systematical analysis of immune infiltrates across diverse cancer types. In this paper, we mainly used it to study the differential expression of HOXB9 between tumor and adjacent normal tissues. The analysis was performed based on the TCGA-UCEC dataset (*n* = 546).

Gene expression profiling interactive analysis, version 2 (GEPIA2) (http://gepia.cancer-pku.cn/) is a web-based bioinformatics tool used for rapid and customized gene set analysis [19]. We initially evaluated the HOXB9 transcription level (TPM) between pan-cancer and corresponding normal tissues. Simultaneously, we analyzed 174 EC tissue samples and 91 normal samples, separately. They were both from TCGA and GTEx database by setting the threshold log2|FC| cutoff = 1 and q-value cutoff = 0.01.

### UALCAN database analysis

UALCAN (http://ualcan.path.uab.edu) [20] provides an interactive web resource and clinical data from the TCGA database of 31 cancer types, which can be used to analyze the relative transcript expression of genes of potential interest between tumor and normal samples. The association between transcript expression and methylation and relevant clinicopathological parameters was analyzed. First, we explored the expression of HOXB9. Thereafter, we analyzed its correlation with multiple clinicopathological parameters, including race, weight, age, menopause status, histological subtypes, TP53 mutation status, and cancer stage. The analyses was performed on data extracted from TCGA_UCEC (uterine corpus endometrial carcinoma, *n* = 546) dataset. In addition, promoter methylation level of HOXB9 was also analyzed based on 46 normal and 438 EC tissues.

### Verification of HOXB9 mRNA expression using qRT-PCR

Tumors and adjacent normal tissues (*n* = 6) were obtained from the Affiliated Hospital of Nantong University. Adjacent normal tissues were separated from the tumor by at least 5 cm. None of the patients received any tumor-related treatment, including radiotherapy or chemotherapy, prior to tissue collection. Tissues were snap-frozen and stored at -80 ℃. The study was approved by the Affiliated Hospital of Nantong University (2022-K155) and performed in accordance with the Declaration of Helsinki and Good Clinical Practice Guidelines. Written informed consent was obtained from all patients.

Specifically, following manufacturer’s instruction, RNA was extracted from snap-frozen tissues using Trizol reagent (Ambion, USA). The mass and concentration of total RNA were measured using a NanoDrop 2000 spectrophotometer. cDNA was synthesized (Vazyme, Nanjing China) from 1 µg of total RNA. The real-time qPCR was performed using SYBR qPCR Master Mix (Vazyme, Nanjing China) in a thermal cycler (Robocycler Gradient 96, BIOMETRA®, Princeton, NJ, USA) to run the reaction. The specific reaction steps were described as follows: pre-denaturation 95℃ for 30 s; cycling reaction: 95℃ for 10 s, 60 ℃ for 30 s, 40 cycles; dissolution curve: 95 ℃ for 15 s, 60 ℃ for 60 s, and 95 °C for 15 s. Relative gene expression was calculated using the 2^−ΔΔct^. Glyceraldehyde 3-phosphate dehydrogenase level was normalized to the mRNA level. Primer information is provided as follows: HOXB9 forward primer, 5ʹ-TAGACTCTTGCTCCTGCTTCTCCTG-3ʹ; HOXB9 reverse primer, 5ʹ-TTTCACGACAGCCACCGACAAAG-3ʹ

### LinkedOmics database analysis

LinkedOmics (http://www.linkedomics.org/login.php) [21] is publicly available portal that includes multi-omics data from all 32 TCGA cancer types and 10 Clinical Proteomics Tumor Analysis Consortium (CPTAC) cancer cohorts. Genes coexpressed with HOXB9 were analyzed statistically and presented in volcano plots and heat maps. We logged into LinkedOmics database, selected "Uterine Corpus Endometrial Carcinoma", searched dataset; selected "HiSeq RNA"; entered gene "HOXB9", selected "Hiseq RNA" for target dataset, selected " Pearson Correlation Coefficient (Pearson test)" for statistical method, submitted, and downloaded the analysis results.

### GO and pathway enrichment analysis

Enrichment analysis of HOXB9 was performed using Enrichr and Metascape. Enrichr (http://amp.pharm.mssm.edu/Enrichr) [22] is a gene set search engine that queries thousands of annotated gene sets. Enrichr uniquely integrates knowledge from many well-known projects to provide comprehensive information on genes and genomes. Metascape (http://metascape.org/gp/index.html) [23] is an effective and efficient tool for the comprehensive analysis and interpretation of omics-based studies in the era of big data. We first established significant genes using Enrichr. HOXB9 was entered in the option box and with ARCHS4 RNA-seq gene–gene co-expression, top 100 genes were identified. Thereafter, using Enrichr and Metascape, pathway enrichment between HOXB9 and 100 co-expression genes were analyzed using three different databases (BioPlanet 2019, WikiPathway 2021 Human , and KEGG 2021 Human). Biological processes (BPs), molecular function (MF), and cell components (CCs) were assessed using GO enrichment analysis. To obtain a more visual effect, 100 co-expression genes were entered into Metascap, human species were selected, and visual results were obtained. The analysis was performed using terms with a *P* value < 0.01, a minimum count of 3, and an enrichment factor > 1.5.

### Protein–protein interaction analysis

As can be seen on the Metascape website, to further capture the relationships between the terms, 100 enriched terms were selected and rendered as a network plot. The network was visualized using Cytoscape (https://cytoscape.org/) [24], where each node represented an enriched term and was colored first by its cluster ID and then by its *P* value. Metascape performed PPI network using four databases, STRING6, BioGrid7, OmniPath8, and InWeb_IM.

### Single-cell RNA-seq analysis

Human Protein Atlas (HPA) (https://www.proteinatlas.org/) [25] is a Sweden-based project founded in 2003 with the goal of mapping all human proteins present in cells, tissues and organs, using various omics techniques. After entering the gene "HOXB9" in the search box, we observed its expression in different single-cell types and tissues, including the endometrium. In HPA, the single-cell RNA sequence of endometrium was derived from the GSE111976 dataset [26].

### cBioPortal database analysis

cBioPortal (https://www.cbioportal.org/) [27] website integrates data from several tumor genomic studies, including somatic mutations, DNA copy number alterations, mRNA and microRNA expression, DNA methylation, protein abundance, and phosphoprotein abundance from TCGA, ICGC, and GEO databases. In addition to information on clinical prognosis, there are other phenotypes in some samples. Using cBioPortal website of visualization platform, 1729 cases of data from five EC datasets (197, 81, 549, 373, and 529 cases) were selected. HOXB9 variants were analyzed from different perspectives using “Cancer Types Summe” and “Oncoprint Options”. In addition, “Survival” module explored the effects of genetic alteration HOXB9 on the prognosis of EC patients.

### Univariate and multivariate Cox regression analysis

Univariate analysis can be used to explore the relationship between predictor variables and OS in patients with EC. However, multifactorial analysis can further exclude the influence of other confounding factors. The agreement between univariate and multivariate analysis indicates that the results are more reliable. In this study, we used the survival package and Cox regression analysis to test proportional risk hypothesis, where samples from single-factor that met a set *P*-value threshold were entered into a multi-factor Cox to build the model.

### Construct a prognostic model

Nomogram, based on multivariate regression analysis, integrates multiple prediction indicators using line segments with scales, and draws them on the same plane according to a certain proportion, so as to express the relationship between various prediction variables in the prediction model. [28]. Based on the results obtained using univariate and multivariate factors, we created a nomogram. Data were downloaded from TCGA database (https://portal.gdc.cancer.gov) and RNAseq was collated using the log2 (value + 1) method to extract data in TPM format as well as clinical prognostic data. These data were tested for proportional risk hypothesis using the survival package[version 3.3.1] and subjected to Cox regression analysis, and the OS nomogram correlation model was constructed and visualized using the rms package [version 6.3–0].

### Assess prognostic predictive power of HOXB9

#### Kaplan–Meier Plotter

Kaplan–Meier plotter (http://Kaplan-Meier-plotter.kmplot.com) [29] is capable of assessing the correlation between the expression of all genes (mRNA, miRNA, protein) and survival in 30,000 samples from 21 tumor types including breast, ovarian, lung, and EC [29].  The sources of the databases include GEO, EGA, and TCGA. The primary purpose of the tool is a meta-analysis-based discovery and validation of survival biomarkers for cancer research. Here, the patients were stratified into low or high expression group, with the median value of HOXB9 set as the cutoff score. KM survival analysis was performed to analyze the prognostic difference between the two groups. The tool was utilized to generate the OS (*n* = 7642) and RFS (*n* = 4420) curves for HOXB9 in UCEC, based on previously described data. Statistically significant differences were considered to exist when *P* value < 0.05.

#### ROC and time-dependent ROC

We analyzed the included variables using the survival package in R studio for Cox regression analysis. RNAseq data of EC were downloaded from TCGA database and collated using log2 (value + 1), data in TPM format were extracted, no clinical information and duplicate data were removed, ROC analysis was performed on the data using the pROC package [version 1.18.0]. Time-dependent ROC analysis was performed on the data using the time ROC package [version 0.4], and the results were analyzed using ggplot2 package [version 3.3.6] for visualization.

### Statistical analysis

In addition to the results automatically generated by public databases, other statistical analyses were performed using R software (version 4.1.3) and GraphPad Prism 8. *P* < 0.05 was considered to be significantly different. One-way ANOVA was use to calculate the difference in specific characteristics between high- and low-risk groups.

## Result

### HOXB9 mRNA up-regulation in pan-cancer

**Fig. 1:**
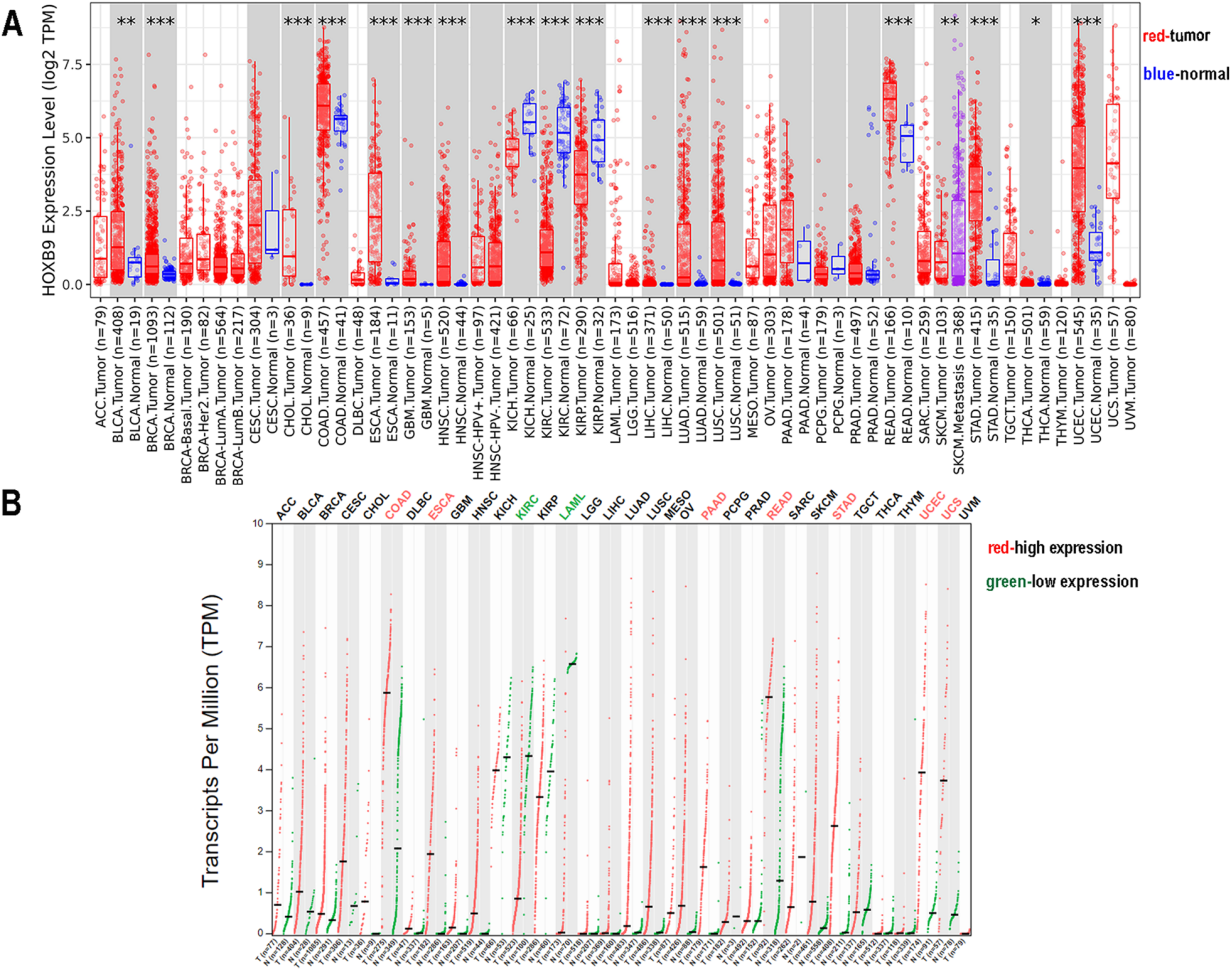
HOXB9 mRNA expression in pan-cancer. **A** HOXB9 expression level by TIMER2.0. **B** HOXB9 expression profile by GEPIA2. (*: P value < 0.05; **: P value < 0.01; ***: P value < *0.001*)

In TIMER2.0 database, differential expression analyses between the tumor and adjacent normal tissues were performed. HOXB9 was significantly higher in UCEC, bladder urothelial carcinoma, breast invasive carcinoma, cholangiocarcinoma, colon adenocarcinoma (COAD), esophageal carcinoma (ESCA), glioblastoma multiforme, head and neck squamous cell carcinoma, liver hepatocellular carcinoma, lung adenocarcinoma, lung squamous cell carcinoma, rectum adenocarcinoma (READ), stomach adenocarcinoma (STAD), and thyroid carcinoma. However, it was significantly lower in kidney chromophobe, kidney renal clear cell carcinoma (KIRC), kidney renal papillary cell carcinoma, and skin cutaneous melanoma (Fig. 1A). For further confirmation, we analyzed and compared the HOXB9 expression using GEPIA2. Of the 33 cancerous tissues, it was significantly higher in UCEC, COAD, ESCA, pancreatic adenocarcinoma, READ, STAD, and uterine carcinosarcoma. However, it was significantly lower in KIRC, acute myeloid leukemia (Fig. 1B). In general, the results were the same for cancer types with high and low HOXB9 expressions in both databases.

### Clinical pathological features and qRT-PCR experimental verification

**Fig. 2:**
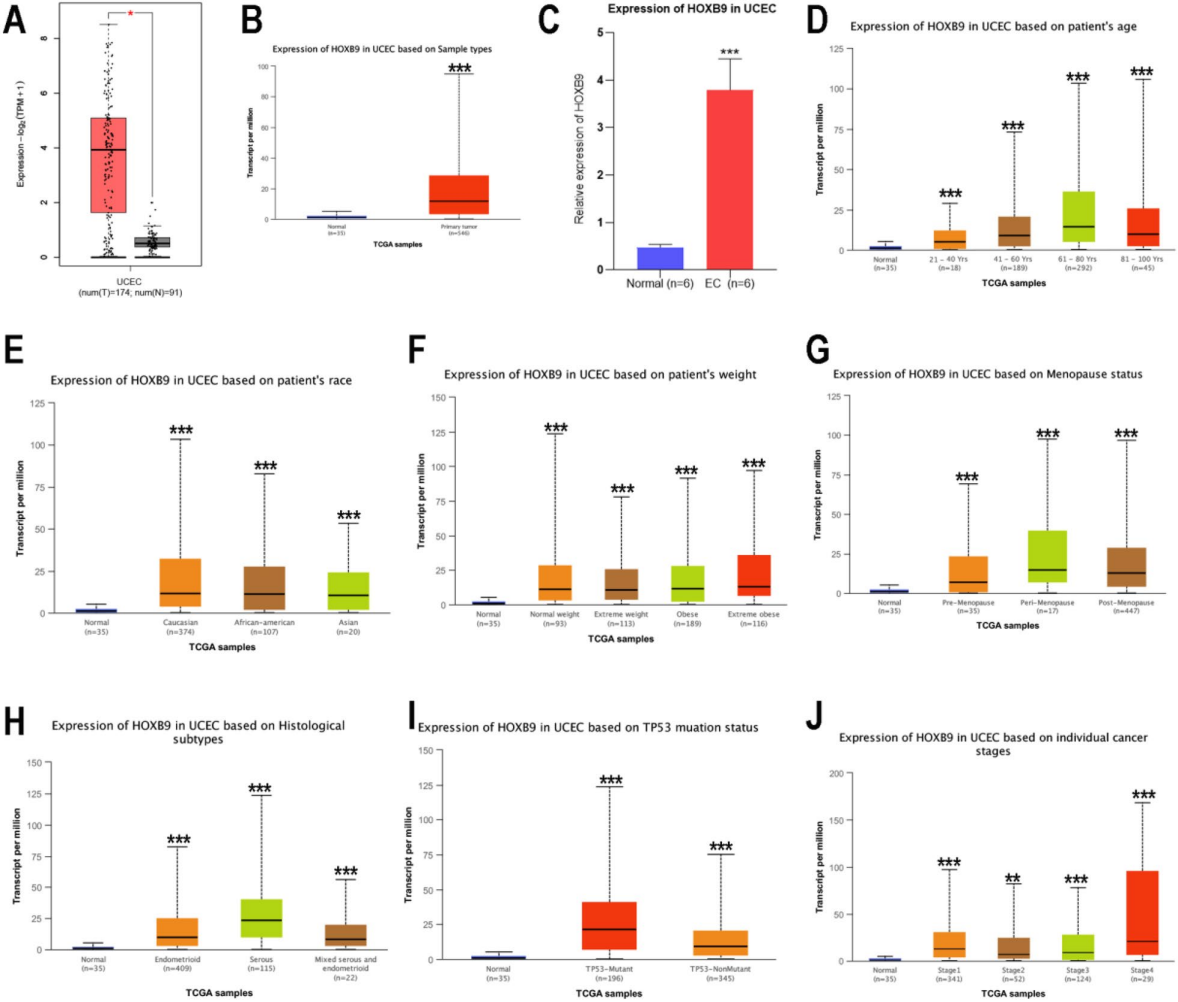
HOXB9 transcriptional level in EC is upregulated. **A** The expression levels of HOXB9 in EC and normal tissues in TCGA by GEPIA2. **B** The expression levels of HOXB9 in tumor tissues and the normal tissues in TCGA by UALCAN. **C** qRT-PCR: expression levels of HOXB9. **D-J:** HOXB9 transcription in subgroups of patients with EC, stratified based on **D** patient’ age, **E** patient’s race, **F** patient’s weight, **G** menopause status, **H** histological subtypes, **I** TP53 mutant status, **J** individual cancer stages. (*, *P* < 0.05; **, *P* < 0.01; ***, *P* < 0.001; ****, *P* < 0.0001)

In another GEPIA2 database, HOXB9 mRNA was significantly and highly expressed in EC tissues (Fig. 2A). The same significant result (*P* = 1.62e−12) was obtained from UALCAN (Fig. 2B). We also verified the expression of HOXB9 in EC samples. qRT-PCR results showed that the expression level of HOXB9 in this tumor was significantly higher than that in adjacent normal tissues (Fig. 2C). Here, the transcription of HOXB9 mRNA was upregulated in EC patients compared with healthy individuals in subgroup analysis based on patient’s age (Fig. 2D), patient’s race (Fig. 2E), patient’s weight (Fig. 2F), menopause status (Fig. 2G), histological subtypes (Fig. 2H), TP53 mutant status (Fig. 2I), and individual cancer stages (Fig. 2J) (*P* < 0.05).

### Co-expression pattern of HOXB9 in EC

**Fig. 3:**
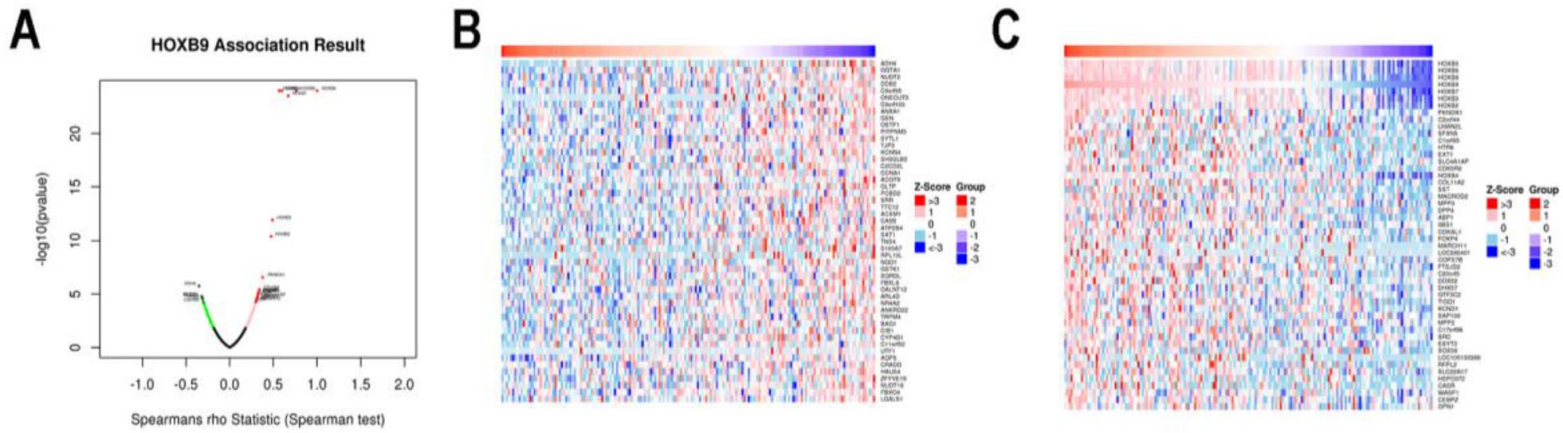
Co-expression analysis results of HOXB9. **A** Volcano map. **B** 50 negatively correlated genes. **C** 50 positively correlated genes (*P* < *0.05*)

To gain insight into its biological significance, we applied the functional module of LinkedOmics to detect the co-expression pattern of HOXB9. As shown in Fig. 3A, dark red dots show a significant positive correlation, while dark green dots show a significant negative correlation (FDR < 0.01). Detailed descriptions of the co-expression genes were shown in Additional file 1. The heat map shows the top 50 significant genes negatively or positively correlated with HOXB9 (Fig. 3B, C).

As shown in Fig. 3C, HOXB9 was strongly correlated  with HOXB8, HOXB7, HOXB6, HOXB5, HOXB4, HOXB3, and HOXB2. Therefore, we speculated the HOX family is involved in the development of EC.

### The role of co-expression genes in EC

**Fig. 4:**
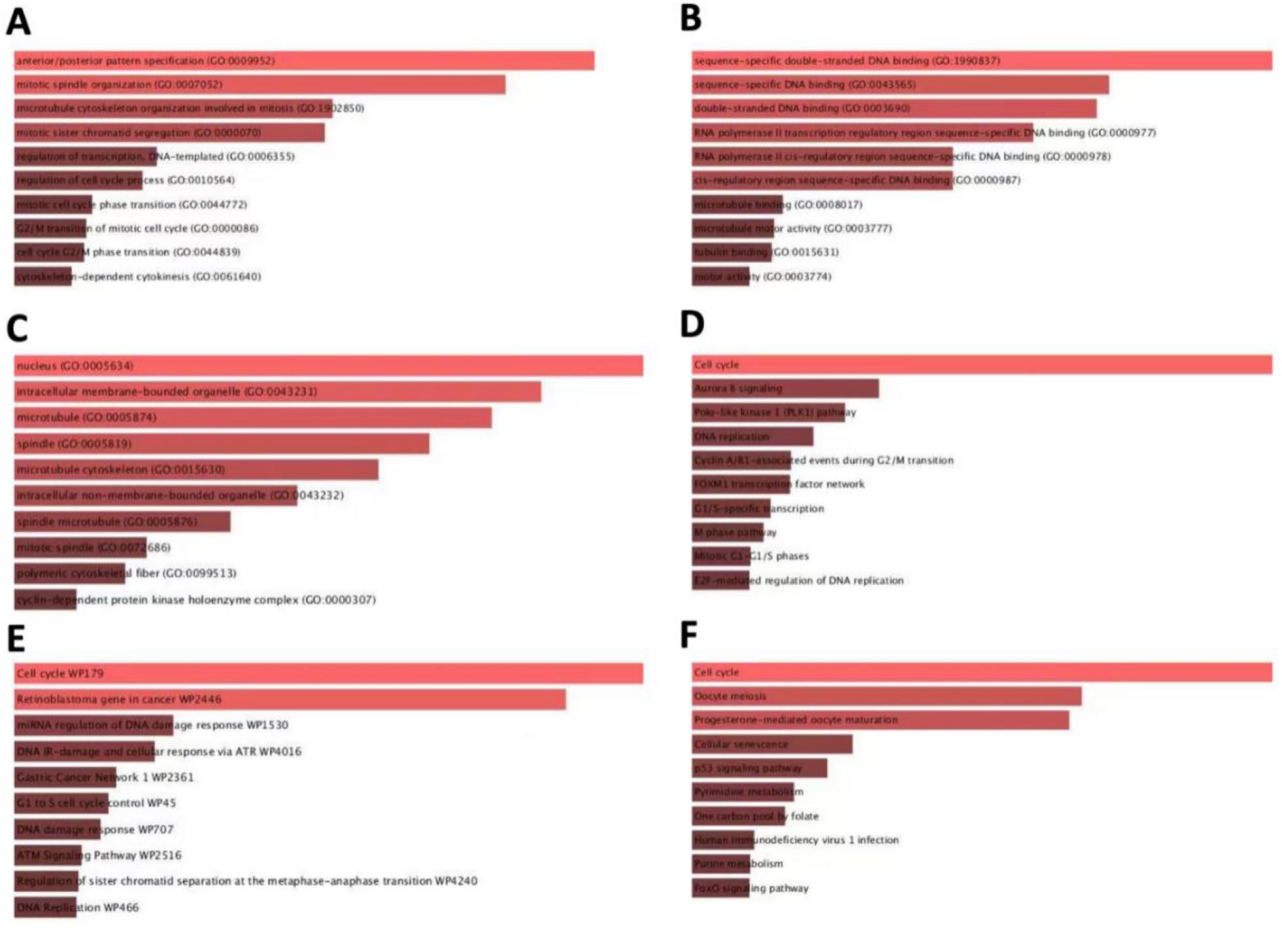
Co-expressed genes profile with the HOXB9 gene involved in GO and signaling pathways in UCEC. **A** GO Biological Process 2021. **B** GO Molecular Function 2021. **C** GO Cellular Component 2021. **D** BioPlanet 2019. **E** WikiPathway 2021 Human. **F** KEGG 2021 Human (*P *< 0.05)

Enrichment analysis was performed to explore the role of HOXB9. Our findings were shown in Additional file 2, featuring 100 co-expression genes. BP enrichment analyses in different database of HOXB9 were mainly associated with mitotic cell cycle-phase transition, G_2_/M transition of mitotic cell cycle, cell cycle G_2_/M phase transition, and cytoskeleton-dependent cytokinesis (Fig. 4A), while MF enrichment analyses were mainly associated with microtubule-binding, micro-tubulin motor activity, tubulin-binding, and motor activity (Fig. 4B). CC was mainly associated with spindle microtubule, mitotic spindle, polymeric cytoskeletal fiber, and cyclin-dependent protein kinase holoenzyme complex (Fig. 4C).

Pathway enrichment analysis demonstrated the involvement of HOXB9 in the FOXM1 transcription factor network, G_1_/S-specific transcription, M phase pathway, mitotic G_1_–G_1_/S phase, E2F-mediated regulation of DNA replication (Fig. 4D); G1 to S cell cycle control, DNA damage response, ATM signaling pathway, regulation of sister chromatid separation at the metaphase–anaphase transition, DNA Replication (Fig. 4E), P53 signaling pathway, pyrimidine metabolism, and FOXO signaling pathway (Fig. 4F).

**Fig. 5:**
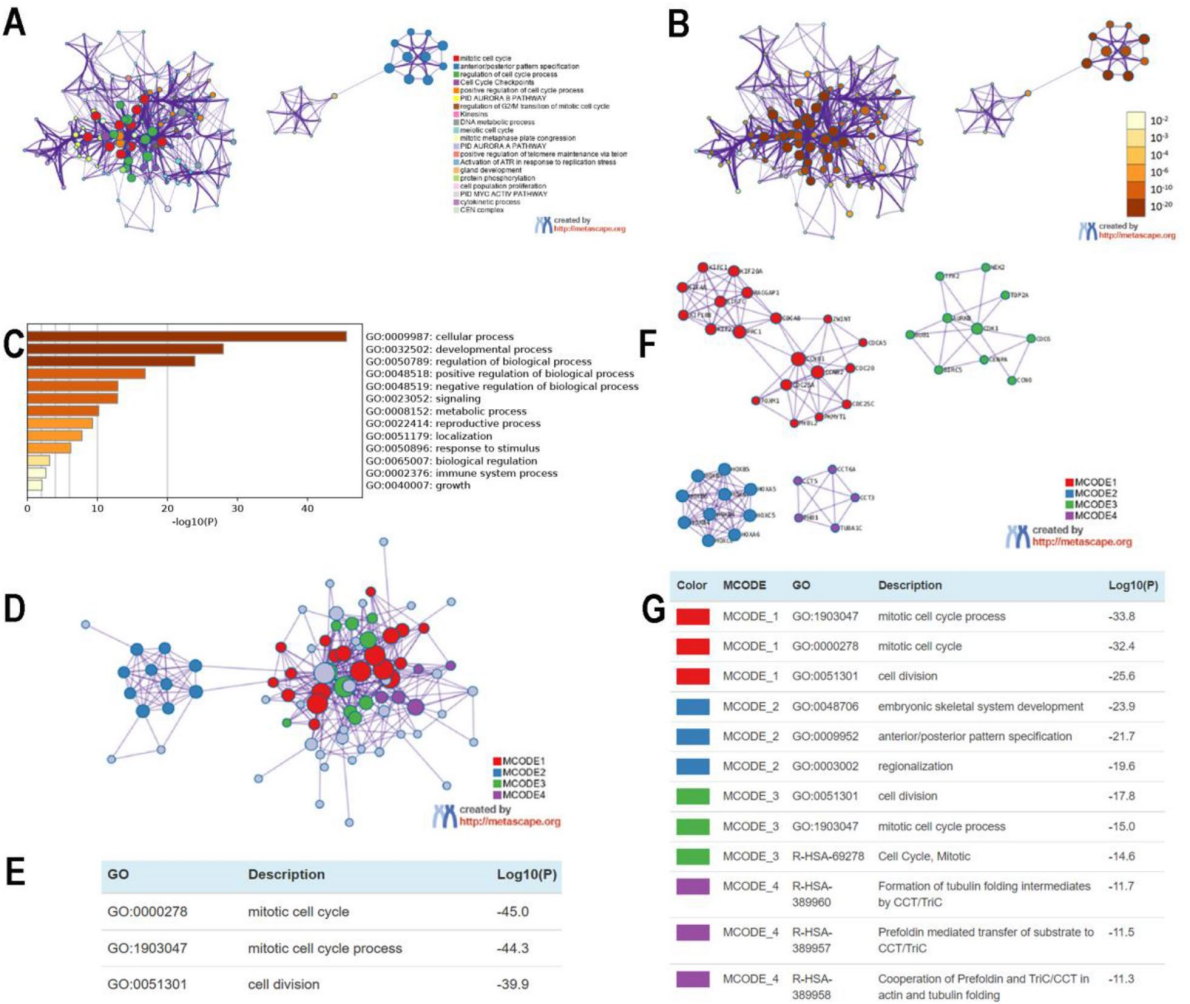
GO and PPI analysis of HOXB9 and co-expression genes in UCEC. Network of enriched terms: ** A** Colored by cluster ID, where nodes that share the same cluster ID are typically close to each other. **B** Colored by *P* value, where terms containing more genes tend to have a more significant *P* value. **C** Bar graph and network of top 20 enriched items in GO analysis. **D**–**G** Protein–protein interaction network and MCODE components in HOXB9 and neighboring genes

Additionally, we validated our results using Metascape. As can be seen on the Metascape website, to further capture the relationships between the terms, 100 genes were selected and rendered as a network plot. After visual analysis using Cytoscape, Fig. 5A, B showed that co-expressed genes were mainly clustered in mitotic cell cycle, anterior/posterior pattern specification, regulation of cell cycle process, cell cycle checkpoints, and positive regulation of cell cycle process.

GO enrichment analysis revealed that HOXB9 was mainly involved in cellular process, developmental process, regulation of biological process (Fig. 5C).

From Fig. 5D-5E, it can be seen that PPI network between co-expression genes is mainly clustered over mitotic cell cycle process, mitotic cell cycle, cell division.

MCODE further revealed that HOXB9 and neighboring genes influenced embryonic skeletal system development, anterior/posterior pattern specification, and regionalization (Fig. 5F-5G).

### Single-cell RNAseq of HOXB9 including endometrium

**Fig. 6:**
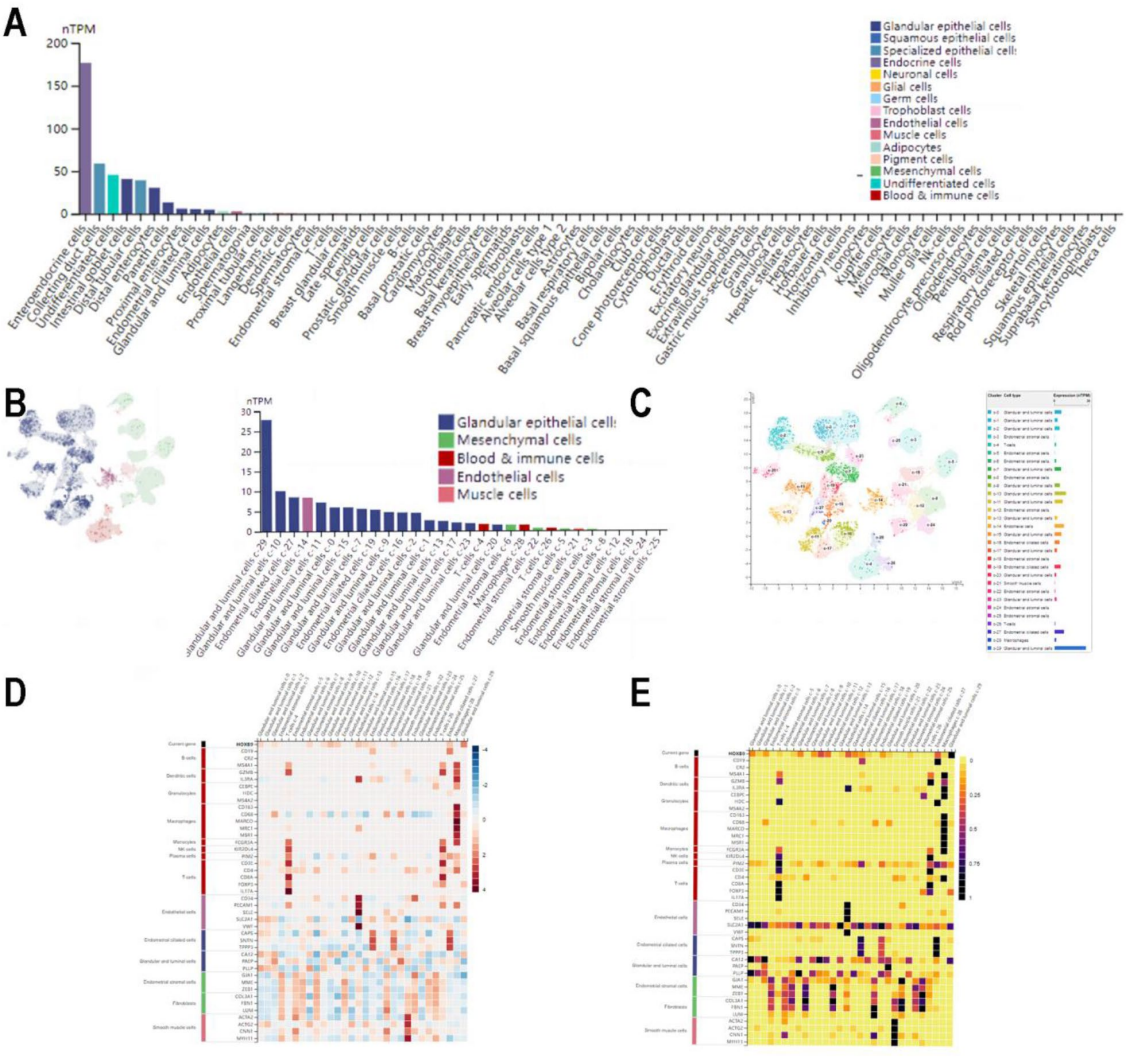
HOXB9 mRNA expression in single-cell types and tissues.** A** An overall survey of different HOXB9 nTPMs in single-cell type specificity. **B** Classification of cells expressing HOXB9 mRNA in the endometrium. **C** Different HOXB9 mRNA expression lever in different cell clusters of endometrium. **D **and** E** Expression of the HOXB9 and type markers in the various single-cell clusters of the endometrium, with two different statistical methods max-norm and Z-score (Enhanced: nTPM ≥ 4, Low specificity: nTPM ≥ 1, Not detected nTPM < 1)

Overall, the top three positions of HOXB9 expression from high to low were entero-endocrine cells, collecting duct cells, and undifferentiated cells. HOXB9 was mainly expressed in entero-endocrine cells, and with 66.1 normalized transcripts per million protein-coding genes (nTPM). Simultaneously, it was also expressed in endometrial-ciliated cells with 4.7 nTPM (Fig. 6A).

In the fraction of single-cell tissues, we found that in addition to the glandular epithelial cells of the endometrium, HOXB9 mRNA also expressed in other cells, including endothelial cells, mesenchymal cells, blood cells, immune cells, and muscle cells (Fig. 6B). Furthermore, each cell type was divided into different cell clusters according to different levels of HOXB9 nTPM expression (Fig. 6C). Using the statistical methods of Mas-norm and Z-score, the heatmap showed expression of HOXB9 and type markers in the various single-cell clusters of the endometrium. We also found that the clusters of cells ranked were glandular and luminal cells c-24, glandular and luminal cells c-9, and endothelial cells c-15, compared with the other cells (Fig. 6D, E).

### Promoter methylation level of HOXB9

**Fig. 7:**
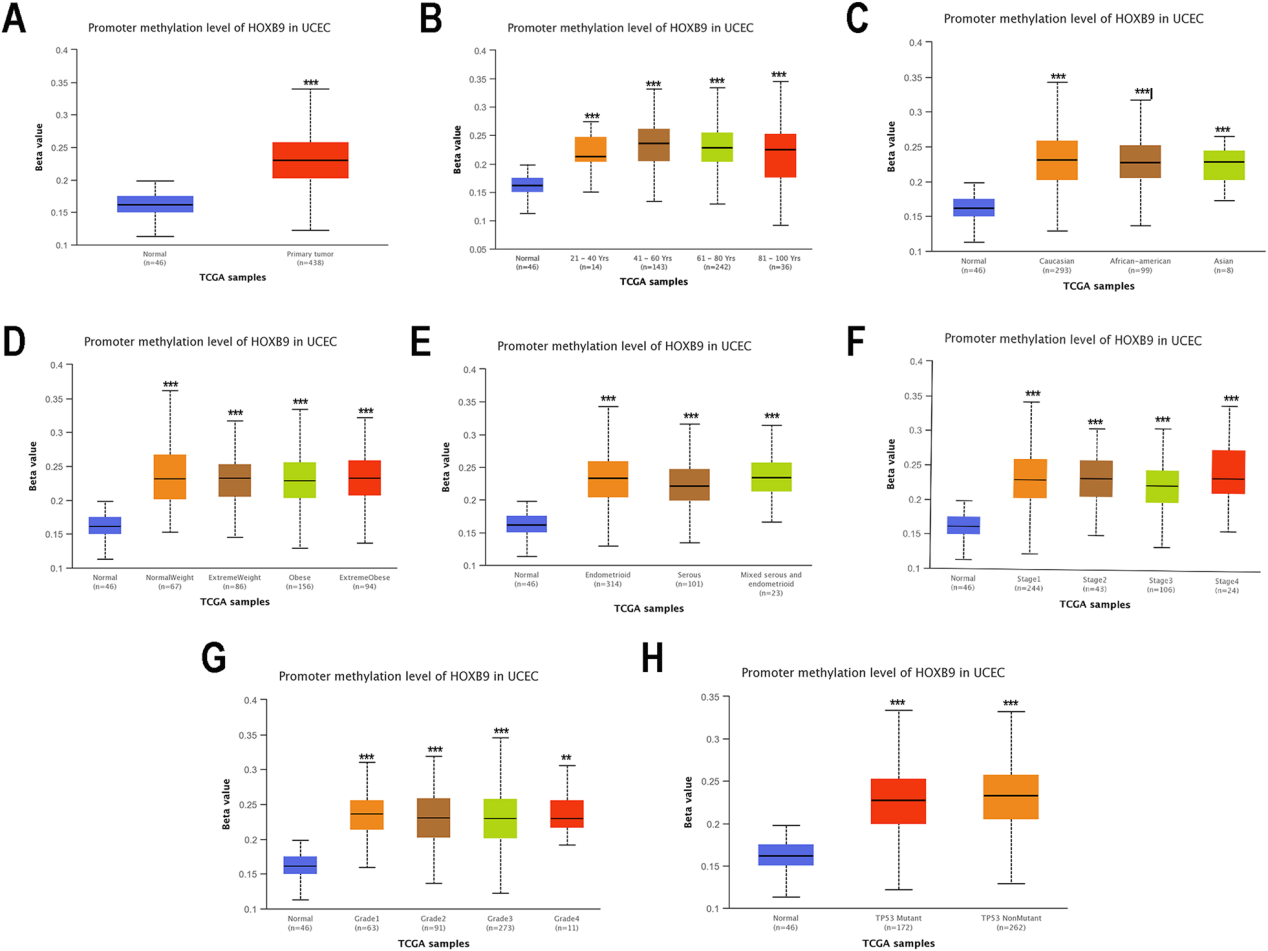
Promoter methylation level of HOXB9 is upregulated in EC. **A** HOXB9 promoter methylation levels of EC patients from TCGA analyzed by UALCAN. **B**–**H**: Subgroup analysis of multiple clinicopathological characteristics about HOXB9 promoter methylation level. **B** patient’s age. **C** patient’s race. **D** patient’s weight. **E** histological subtype. **F** individual cancer stage. **G** tumor grade. **H** TP53 mutation status (*: P value < 0.05; **: P value < 0.01; ***: P value < *0.001*)

DNA methylation, resulting in the genetic alteration, is well-studied epigenetic change and plays an important role in the initiation and progression of carcinogenesis [30]. As expected, promoter methylation levels of HOXB9 were significantly higher in primary tumors than in normal tissues, as shown in the UALCAN TCGA-EC database (Fig. 7A). Further subgroup analysis of multiple clinicopathological characteristics showed consistent upregulation of HOXB9 promoter methylation level according to patient’s age (Fig. 7B), patient’s race (Fig. 7C), patient’s weight (Fig. 7D), histological subtype (Fig. 7E), individual cancer stage (Fig. 7F), tumor grade (Fig. 7G), and TP53 mutation status (Fig. 7H).

### Alterations of HOXB9 in EC

**Fig. 8:**
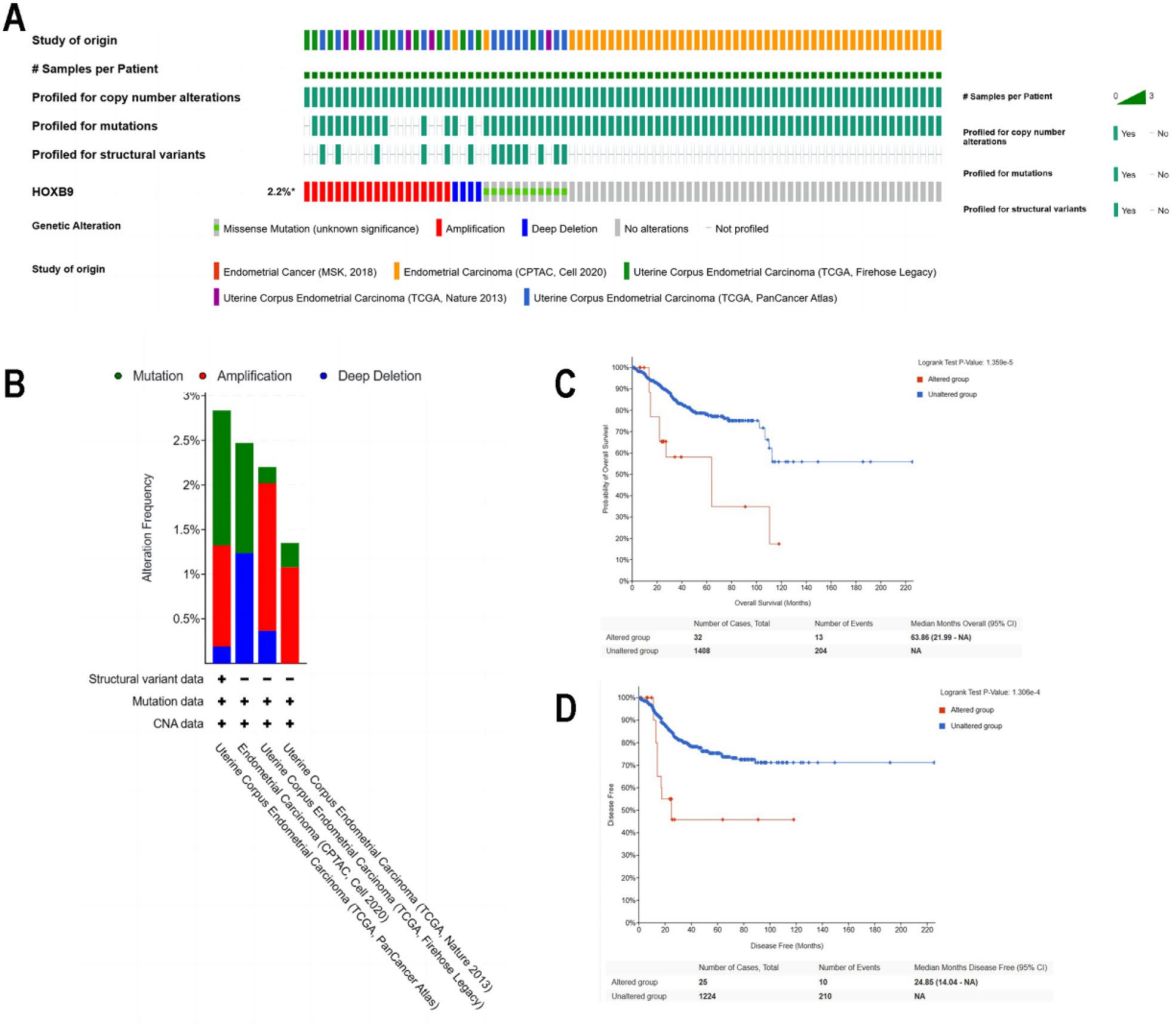
Alteration of HOXB9 gene in EC and the effect on the prognosis of EC patients. **A** Waterfall plot for the distribution and classification of genetic alteration in HOXB9 in UCEC. **B** HOXB9 variants in different EC datasets. **C** The overall survival curve between altered and unaltered group. **D** The disease-free survival curve between altered and unaltered groups

As genetic mutations are associated with poor prognosis in cancer patients [31–33], we analyzed the gene alteration of HOXB9 in EC samples from the cBioPortal database. The results are as follows. In the Oncoprint section, there were 1526 EC samples with complete gene sequence and copy number variation information, among which 34 cases had gene mutation, and the total mutation rate was 2.2% (34/1526), including four cases of fusion deep deletion, 11 cases of missense mutation, and 19 cases of amplification (Fig. 8A). Genetic alteration analysis revealed that the genetic alteration in HOXB9 were mainly amplification and missense mutation.

Overall, in the Cancer Types Summer section, four of five categories (Cancer Study) were shown based on filtering. Figure 8B shows the alteration frequency of HOXB9 in different datasets, as well as the ratio of mutation, amplification, and deep deletion composition. In the dataset of Uterine Corpus Endometrial Carcinoma (TCGA, PanCancer Atlas), HOXB9 was altered in 2.84% of 529 cases. In the Endometrial Carcinoma dataset (CPTAC, Cell 2020), HOXB9 was altered in 2.47% of 81 cases, whereas in the Uterine Corpus Endometrial Carcinoma dataset (TCGA, Firehose Legacy), HOXB9 was altered in 2.2% of 545 cases. In the dataset of Uterine Corpus Endometrial Carcinoma (TCGA, Nature 2013), genes altered in 1.35% of 371 cases.

Follow-up analysis was performed to explore the correlation between HOXB9 and the major hallmarks of EC using the survival module of cBioPortal. Interestingly, these genetic alterations had the effect on the OS (Fig. 8C, *P*= 1.359e−5) and disease-free survival (Fig. 8D, *P* = 1.306e−4) in patients with EC.

### Univariate and multivariate Cox regression analysis

**Fig. 9:**
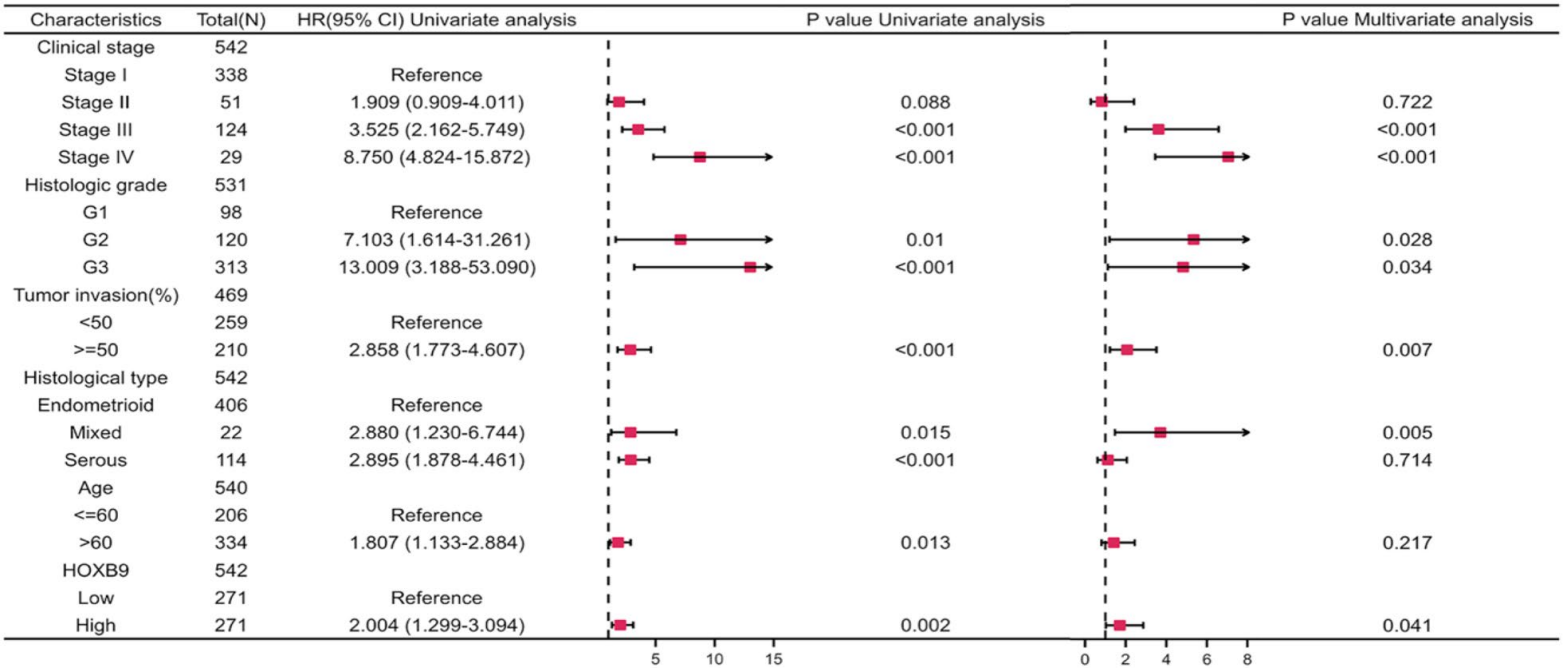
Univariate and multivariate Cox regression analysis of HOXB9 and other clinicopathologic parameters with OS

To further evaluate whether HOXB9 and other risk factors had an prognostic predicting function, univariate and multivariate Cox regression analyses were performed. The results showed a significant relationship between HOXB9 mRNA expression and six clinicopathological parameters that were closely correlated with OS in patients with EC. They were stages III and IV, G2 and G3, tumor invasion ≥ 50%, mixed or serous histological type, age > 60 years, and high expressions of HOXB9. The detailed HRs for each parameter were in Fig. 9.

These analyses confirmed the prognostic relevance of HOXB9. Finally, we took full advantage of these results to construct a prognosis-related nomogram.

### Nomogram

**Fig. 10:**
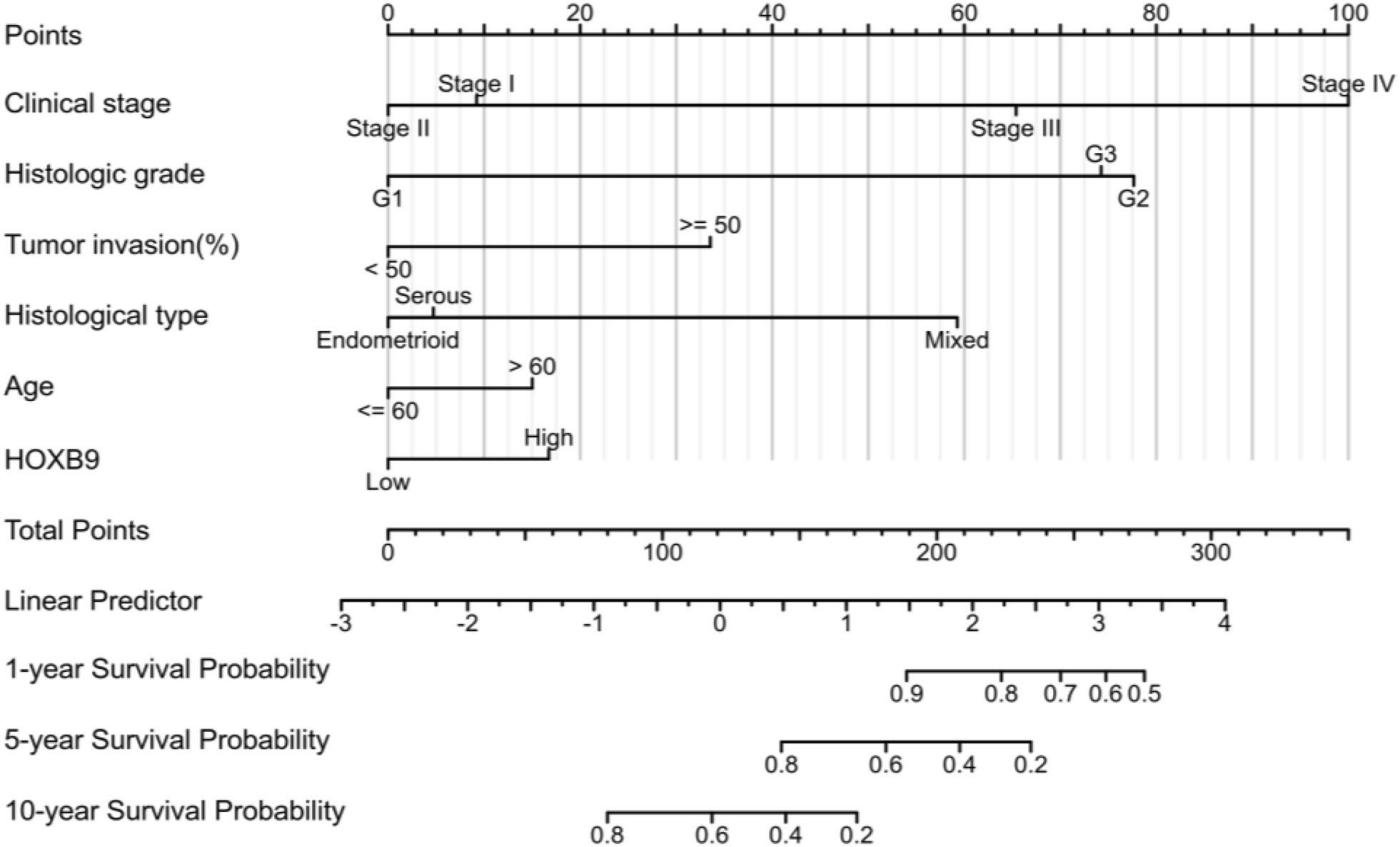
Prognostic nomogram of 1-, 5-, and 10-year

Based on the above results, we established a model. Six factors were incorporated into the nomogram. The relative 1-year, 5-year, and 10-year survival rates were determined (Fig. 10).

### Verification of a prognostic model for EC patients

**Fig. 11:**
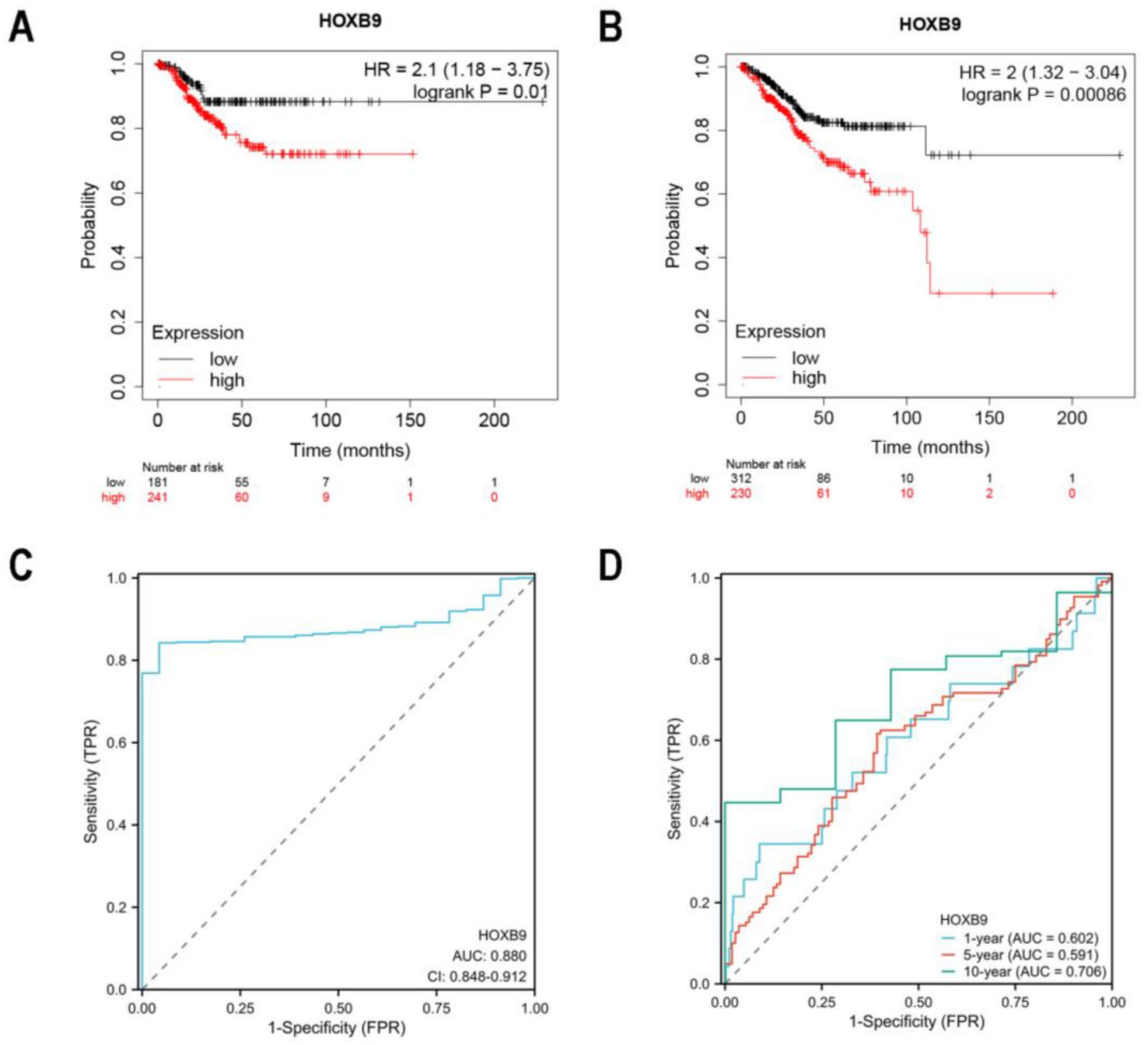
KM survival plots and ROC curves. **A** Relationship between the expression of HOXB9 and RFS. **B** Relationship between expression of HOXB9 and OS. **C** ROC. **D ** time-dependent ROC

The data obtained from the Kaplan–Meier plotter showed that patients were sub-classified into low- or high-risk groups based on the median risk score. The OS of low HOXB9 expression cohort was 111.63 months, while that of high HOXB9 expression cohort was 41.63 months. The results showed EC patients overexpressing HOXB9 (HR = 2.1, 95%:1.18–3.75, log rank *P* = 0.01) had a worse RFS (Fig. 11A). In addition, regarding OS, the result was the same as a worse OS (HR = 2, 95%:1.32–3.04, log rank *P* = 0.00086) (Fig. 11B).

The ROC graph reflects the relationship between sensitivity and specificity. It is often used to evaluate diagnostic tests. The AUC generally ranges between 0.5 and 1, the closer the AUC is to 1, the better the variable is in predicting outcome. Figure 11C shows the AUC was as high as 0.880, and the AUCs of time-dependent ROC were 0.602, 0.591, and 0.706 for 1-year, 5-year, and 10-year survival probabilities, respectively (Fig. 11D). Therefore, HOXB9 could be a potential prognostic biomarker for UCEC.

## Discussion

HOXB9 plays a crucial role in many human solid cancers, and its aberrant expression significantly contributes to the tumor formation [34, 35]. High levels of HOXB9 are associated with a poor prognosis in lung adenocarcinoma patients [36], low overall survival in colon cancer [37], high cancer grade but low overall survival in breast cancer [38], clinical progression in glioma patients [39], gastric cancer tumor progression, vascular and lymphatic invasion [40], and low vascular invasion and overall survival in hepatocellular carcinoma patients [41]. However, HOXB9 downregulation was also reported to be associated with a poor survival in gastric cancer patients, highlighting conflicting hypotheses regarding the role of HOXB9 in cancer [42]. In our pan-cancer analysis, we found HOXB9 expression was elevated in these cancers, with roughly the same results.

By analyzing the expression of HOXB9 in various subgroups, we obtained several interesting results. The transcription of HOXB9 mRNA was upregulated compared with healthy individuals. The HOXB9 expression level was the highest in the extreme obese group, which also coincided with obesity as a high-risk factor for EC. From the individual cancer stages analysis, HOXB9 expression was higher in stage IV than in all other stages, suggesting that high HOXB9 expression is a poor prognostic factor. According to pathological classification, HOXB9 had the highest expression level in serous pathological types. Based on TP53 mutation status, HOXB9 expression was more pronounced when TP 53 mutated, suggesting that TP 53 may be a high-risk factor for EC.

Over the past decades, emerging studies have shown the regulation of multiple biological processes, such as biological signaling, regulation of gene expression, energy and substance metabolism, and cell cycle regulation, depends on PPI network, not just individual proteins [43]. Wan et al. found HOXB9 promotes EC progression by targeting E2F3. The result also showed that E2F3 knockdown abolished the ability of HOXB9 to enhance cell migration [44]. In this study, we used LinkedOmics and Enrichr to identify the genes co-expressed with HOXB9 and explored the interactions. PPI network analysis showed that HOXB9, HOXB8, HOXB7, HOXB 6, HOXB 5, HOXB 4, HOXB 3, and HOXB2 had the potential to interact. The results suggested these proteins may exert their function together and have similar functions in the EC.

Pathway enrichment analysis showed HOXB9 and its co-expressed genes are associated with the regulation of multiple signaling. For instance, G_1_/S-specific transcription, M phase pathway, Mitotic G_1_–G_1_/S phase, E2F-mediated regulation of DNA replication [45], G1 to S cell cycle control, DNA damage response, and P53 signaling pathway. Zhan and Chiba et al. studied HOXB9 expression to mediate angiogenesis, EMT and tumor stem cell properties through the TGF-β pathway, leading to chemoresistance and poor overall prognosis in pancreatic cancer [46, 47]. Zhan et al. also found that Kindlin-2, induced by TGF-β signaling, promoted the progression of pancreatic ductal adenocarcinoma by downregulating the transcription factor HOXB9 [46]. These results suggest that HOXB9 is involved in the development of various tumors via signal transduction.

Single-cell RNA sequencing is a new technique which can analyze the characteristics of various tumor and other cells in the tumor microenvironment to more accurately explain the mechanism of oncogenes, improve the diagnostic efficiency of tumors and the level of personalized treatment, and predict tumor outcomes [48–50]. In patients with high-grade serous tubo-ovarian cancer, single-cell RNA-seq identifies the stromal cell phenotype that can predict OS, is a promising approach to predict prognosis or therapy response [50]. Park et al. characterized 57,979 cells from healthy mouse kidneys using unbiased single-cell RNA sequencing. Based on gene expression patterns, they inferred that inherited kidney diseases caused by different gene mutations but with the same phenotypic expression originate from the same differentiated cell type [51]. Tirosh et al. analyzed malignant, immune, mesenchymal, and endothelial cells using single-cell RNA sequencing (RNA-seq). Non-malignant cells were pooled according to cell type, the relationship between non-malignant tumor cells (e.g. CAFs) and malignant gene expression patterns and drug sensitivity was further determined, and CAF genes associated with T cell infiltration were identified [52]. In this study, we discovered high expression of HOXB9 in the endometrium. We speculate that the expression of HOXB9 in specific cell types is related to EC differentiation status and drug sensitivity but requires further investigation. We will therefore present the significance of HOXB9 in the endometrium in our future work.

With intensive research in genomics, the effects of epigenetic alterations on tumorigenesis have gradually attracted the attention of researchers. As the most common and critical epigenetics modification, dysregulation of DNA methylation is considered a key factor leading to the carcinogenesis of various tumors, including EC [53]. Several genes with specific hypermethylation or hypomethylation have been identified in several cancers [54]. Our study showed HOXB9 promoter methylation was associated with EC, and also found the HOXB9 variant was closely associated with OS and RFS, and OS and RFS were significantly lower than those in the unaltered group.

Based on the consistent results obtained by univariate and multivariate Cox regression analysis, it is reasonable to think the high expression of HOXB9, high stage, high grade, tumor invasion greater than 50%, and histological type, and age greater than 60 years jointly affect the OS in EC patients. Therefore, we constructed the nomogram, KM curves, diagnostic ROC and time-dependent ROC analysis verified the predictive performance.

Although our study employed rigorous bioinformatics and statistical methods to determine the prognostic value of HOXB9 expression in EC patients, it still has certain limitations. First, only normal endometrium was discovered in the single-cell database, and EC tissue could not be found. Second, no additional experiments were performed to analyze our results, other than the qRT-PCR validation of HOXB9 expression in clinical samples.

## Conclusion

In conclusion, our study showed HOXB9 plays a crucial role in the tumorigenesis and development of EC. The constructed nomogram model could better predict the prognosis of EC.

## Supplementary Information

**Additional file 1.** Correlated genes of HOXB9

**Additional file 2.** Top 100 genes co-expressed with HOXB9

## Data Availability

Publicly available datasets were analyzed in this study. Data are available at the TCGA (https://portal.gdc.cancer.gov/), HPA (https://www.proteinatlas.org/), GEPIA2 (http://gepia.cancer-pku.cn/), TIMER2.0 (http://timer.cistrome.org/), UALCAN (http://ualcan.path.uab.edu/index.html), c-BioPortal (https://www.cbioportal.org/), LinkedOmics (http://linkedomics.org/login.php), Enrichr (https://maayanlab.cloud/Enrichr/), Metascape (http://metascape.org/gp/index.htm).

## Abbreviations

EC: endometrial carcinoma
UCEC: uterine corpus endometrial carcinoma
qRT-PCR: quantitative real time polymerase chain reaction
OS: overall survival
RFS: recurrence free survival
ROC: receiver operating characteristic
AUC: area under curve
KM: Kaplan-Meier
nTPM: normalized transcripts per million
HR: hazard ratio
GO: gene ontology
KEGG: Kyoto Encyclopedia of Genes and Genomes
PPI: protein-protein interaction
BP: biological processes
MF: cellular molecular
CC: cell component
COAD: colon adenocarcinoma
ESCA: esophageal carcinoma
READ: rectum adenocarcinoma
STAD: stomach adenocarcinoma
KIRC: kidney renal clear cell carcinoma
